# Purification and Characterization of Pinto Bean Protein Using Membrane Technology

**DOI:** 10.1002/fsn3.4511

**Published:** 2024-11-19

**Authors:** Neda Aliabbasi, Levente L. Diosady, Zahra Emam‐Djomeh

**Affiliations:** ^1^ Transfer Phenomena Laboratory (TPL), Department of Food Science, Technology and Engineering, College of Agriculture and Natural Resources University of Tehran Karaj Iran; ^2^ Department of Chemical Engineering and Applied Chemistry University of Toronto Toronto Ontario Canada

**Keywords:** diafiltration, extraction, membrane processes, pinto bean protein, ultrafiltration

## Abstract

Pinto beans, an underutilized legume, are abundant in protein content and contain a variety of beneficial phytonutrients. However, the commonly used protein extraction method, alkaline extraction, is associated with several drawbacks. These drawbacks include low extraction yield and purity as well as the production of large amounts of wastewater that can lead to environmental hazards. In this regard, membrane technology has gained considerable recognition as a superior method for extracting proteins. A combined processing scheme was developed, which included alkaline extraction at pH 10.5, ultrafiltration with a concentration factor of 5.5, diafiltration with a diavolume of 4, and isoelectric precipitation at pH 4.5 followed by freeze drying. The specific functional characteristics (nitrogen solubility index, water and oil holding capacity, and emulsifying and foaming properties) of the protein concentrates were assessed and compared with those of a commercially available soybean protein isolate. Based on pinto bean flour containing 23.9% protein, 85.5% of the protein was recovered in the products of this process: precipitated protein concentrate (PPC) with 86.4% protein, acid‐soluble protein concentrate (ASP‐C) with 56.3% protein, and meal residue with 6.1% protein. The mass yields were 17.3% in PPC, 3.9% in ASP‐C, and 54% in the meal residue. The precipitated protein showed higher emulsifying activity, and the acid‐soluble protein showed a high nitrogen solubility index (NSI) and oil‐holding capacity. Both proteins had comparable foaming properties to commercial soy protein isolate. The project demonstrated the feasibility of protein production from pinto beans and highlighted the proteins' useful food functionality and good potential for commercialization.

## Graphical abstract

Two protein products PPC and ASP‐C with a protein content of 86.4% and 56.3% and the yield of 17.3% and 3.9% was successfully produced. The optimal conditions for recovering pinto bean protein via membrane processing were determined to be a pH of 10.5 for extraction, pH of 4.5 for precipitation, concentration factor of 5.5 for concentrating, and diavolume of 4 for purification.

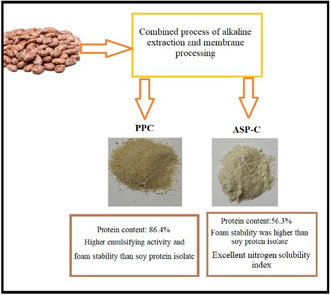

## 1 Introduction

Plant proteins play a vital role in the human diet, particularly in developing nations where the average protein consumption falls below the recommended levels. The scarcity of sufficient animal protein resources has generated a strong interest in discovering alternative plant‐based protein sources that offer similar nutritional and functional benefits in food systems (Chasquibol et al. 2022; Kanu et al. 2007). Plant‐based protein products have typically been derived from soy protein, which is widely available in the global market as a rich source of dietary protein. However, the need for affordable plant‐based protein options and the desire for environmental and health advantages motivate the food industry to explore plant proteins with functional properties that can replace soy protein (Arora et al. 2023; Aschemann‐Witzel et al. 2021). Common beans have a significant impact by providing essential protein (20%–30%) and complex carbohydrates (55%–75%), and their affordability and essential nutrients make them a staple food in developing countries (Mecha et al. 2021). Common beans have not been subjected to genetic modification (Ferawati et al. 2021). Moreover, numerous studies have demonstrated the positive effect of consuming beans in preventing diseases like type 2 diabetes, stroke, and various forms of cancer (Messina 2014). The pinto bean belongs to the common bean family (*Phaseolus vulgaris* L.) and is one of the most popularly cultivated and consumed bean varieties in the United States (Câmara, Urrea, and Schlegel 2013). The pinto bean, a legume that is often overlooked, contains abundant protein and phytonutrients (Ferreira et al. 2022). According to Anton et al. (2009), pinto beans are high in fiber (15 g/100 g) and protein (23 g/100 g) and low in fat (1 g/100 g). Furthermore, the World Health Organization acknowledges and recognizes it as a reliable source of protein with no considerable amount of fat (Ngoh and Gan 2016). The main protein components of pinto beans are vicilin, with a molecular weight of 41 kDa, and phytohemagglutinins, which come in various sizes (30, 29, 27, 26, 23, and 16 kDa) (Tan, Ying‐Yuan, and Gan 2014). Vicilin, the main stotage protein in phaseolus beans, is composed of three smaller units (α, β, and γ phaseolin) and the molecular weight of the substance falls within the range of 43–53 (kDa). It does not contain any disulfide bonds (Van Kleef 1986). Phytohemagglutinins possess the ability to bind to sugars and can cause hemagglutination, but high doses of this fraction can be toxic to animals (Nasi, Picariello, and Ferranti 2009). Additionally, in SDS‐PAGE analysis, it has been observed that pinto beans contain 11S legumin subunits (195 and 9 kDa), along with a few less prominent bands with higher molecular weights (55, 76, and 98 kDa) (López‐Pedrouso et al. 2012). Functional properties refer to the physical and chemical attributes of proteins that impact their performance in various stages of food handling, including processing, storage, preparation, and consumption. These traits may be influenced by elements like the protein's source as well as the methods used for flour processing and the extraction and production of protein concentrates and isolates (Gerliani, Hammami, and Aïder 2020; Su and Xu 2015). Moreover, physiochemical factors such as pH, temperature, salt concentration, and ionic strength can greatly influence the functional characteristics of proteins (Arogundade et al. 2006; Fasuan, Gbadamosi, and Omobuwajo 2018; Oroumei, Rezaei, and Chodar Moghadas 2024; Ozgolet et al. 2024). Good functional properties and acceptable organoleptic properties are required for a protein isolate to qualify as a component in food. In previous studies, pinto bean protein was extracted by alkaline solubilization and acid precipitation and was found to have significantly higher amounts of lysine and better thermal properties and emulsifying activity (EA) than soy protein isolates (Tan, Ying‐Yuan, and Gan 2014). High‐quality proteins can be recovered through modification of a membrane‐based process which was initially developed for rapeseed protein (Xu and Diosady 1994). The process of membrane‐based protein extraction involves several stages: protein solubilization using an alkaline solution, precipitation at the isoelectric point, purification, and subsequent drying (Ozgolet et al. 2024). The final products are recovered in the form of protein concentrates (> 65% protein) and/or isolates (> 90% protein) on a dry basis (Boonlao, Ruktanonchai, and Anal 2023). The membrane‐based technique has been employed for protein extraction, resulting in the production of three products that exhibit remarkable protein recovery from various oil seeds (Diosady 1994; Kumar et al. 2020; Marnoch and Diosady 2006; Sarv, Trass, and Diosady 2017; Tzeng, Diosady, and Rubin 1990). Kumar et al. (2020) produced precipitated and soluble protein isolates through membrane processing which contained ~92% and ~62% protein on a dry basis, respectively. Tzeng, Diosady, and Rubin (1990) optimized membrane technology to produce high purity protein isolates containing 87%–104% protein free of glucosinolates. Marnoch and Diosady (2006) modified membreane parameters to develop protein products where the protein isolate (PPI) showed similar quality to soy protein isolate when added to typical meat products, with regards to characteristics like color, texture, and taste. In the study conducted by Kim, Liceaga, and Yoon (2019), the proteins from perilla seed by‐products (PSM) were enzymatically digested with trypsin to produce antioxidant peptides, and the resulting PSM protein hydrolysate was subsequently purified using ultrafiltration. The purified peptides showed strong antioxidant properties, including reducing capacity, DPPH radical scavenging ability, and ABTS radical scavenging capability. Due to the presence of several undesirable and anti‐nutritional factors, such as glucosinolates, phenolics, and phytates, the utilization of legume proteins has been limited (Bora 2014). According to Marnoch and Diosady (2006), membrane processing can effectively decrease the levels of glucosinolates and phytates, as they are small molecules and can easily pass through the membrane. However, phenolic compounds cannot be removed efficiently since certain compounds have the ability to bind with proteins in water‐based environments, resulting in the formation of phenolic‐protein conjugates. The dark color and the strong bitter flavor of protein isolates are attributed to the presence of these phenolic‐protein complexes (Marnoch and Diosady 2006). The process of protein extraction using membrane technology was modified by Xu & Diosady (1994) to lower the phenolics content of the protein‐based products. The protein products had high protein content with good food functionality, while the glucosinolate and phytate content in these products was reduced significantly. In the patented process described by Diosady, Xu, and Chen (2005), an oilseed flour is solvent extracted to remove oil, and proteins are extracted by aqueous alkali. The phenolic complexes of alkaline extracted proteins are dissociated, and the released phenolics are removed by membrane processing. The protein solution is concentrated and purified, and then, an isoelectric protein is produced by acid precipitation. The acid‐soluble proteins are further concentrated by membrane processing and dried. In the case of legume proteins, initial defatting is not needed due to their low oil content, and their flour can be used directly for protein extraction. Protein products produced by membrane processing provide superior functional properties when compared to those produced using traditional methods. Although conventional extraction techniques can reduce the presence of oligosaccharides in protein concentrates, they may still contain notable quantities of phytic acid and insoluble carbohydrates (Omosaiye and Cheryan 1979). Furthermore, a portion of proteins can be lost during the production process, leading to a decrease in overall protein yield. To overcome these challenges and attain superior quality products, the application of membrane technology has been proposed for the manufacturing of protein concentrates. Several studies have emphasized the improved functional properties of protein concentrate and isolate processed using membranes in comparison to traditional acid‐precipitated products (Boye et al. 2010; Lopes Lorenzetti 2021). Membrane separation technology offers unique advantages compared to conventional separation and purification methods. These advantages include high separation efficiency, environmental sustainability, simplified filtration processes, and effortless control. To the best of our knowledge, there are no studies on the potential of membrane technology for extracting protein from pinto beans. Therefore, the objective of this study was to use the membrane‐based method in order to establish an efficient process for eliminating nonprotein substances to maximize the recovery of protein and increase the protein content in the final product. Initially, the impact of pH on the extractability of proteins was assessed. Based on the results, optimal pH values were selected to extract and precipitate protein, and the applicable concentration factor (CF) and diavolume (DV) for ultrafiltration and diafiltration were determined. The protein content of both the pinto beans and the protein products including precipitated protein concentrate (an isoelectric protein isolate), acid‐soluble protein concentrate (a soluble protein isolate), and Meal residue (the remaining solid material after oil and protein extraction) generated from them were assessed, to calculate the protein recovery. Furthermore, the functional characteristics (the nitrogen solubility index, water, and oil holding capacity, emulsifying activity and stability, whippability, and foam stability) of the membrane pinto bean protein concentrates were compared to the commercial soy protein isolate.

## 2 Materials and Methods

An aqueous 0.5 g/mL solution of sodium hydroxide and 0.85 g/mL phosphoric acid were purchased from Sigma Millipore (Toronto, Canada) and diluted to the required concentration. Sodium chloride and all of the other required compounds were purchased from Fisher Scientific (Fairlawn, NJ) and BDH (Toronto, Canada). A hot plate stirrer was used for heating and mixing samples. Dry pinto beans were obtained from Bulk Mart (Toronto, Ontario, Canada). The beans were dehulled manually, for this purpose; first, beans (10 kg) were soaked in fresh water and maintained at ambient temperature for a duration of 12 h. Then, they were rubbed between the palms to extract the hulls followed by drying under a fume hood for 3 days (Mang et al. 2015). The pinto bean flour was prepared by grinding via a laboratory grinder mill (CM 290 Cemotec, Denmark) and passing through a 45 US mesh sieve (0.355 mm openings). Commercially available soybean protein isolate (SUPRO 500E) with a protein content of 92.0% (dry basis) (*N* × 6.25), a maximum ash content of 5 wt.%, and a maximum moisture content of 6 wt.% was obtained from protein technologies international, based in St. Louis, MO, USA, and was used as the standard. A SEPA CF II (Sterlitech Corporation, Washington USA) ultrafiltration unit was used for both the ultrafiltration and diafiltration. According to the methods of Tzeng, Diosady, and Rubin (1990), the solution was drawn from a feed container using a peristaltic pump and pumped through the UF membrane (Polysulphone, Sepa Cell Membranes) possessing a molecular weight cut‐off of 10,000 D and a membrane surface area of 0.1 m^2^. The permeate, which contained water and impurities in the form of dissolved low molecular weight compounds, was gathered and ultimately disposed of as waste. Throughout the purification procedure, the flow rate of the permeate was controlled by means of a back‐pressure valve positioned at the outlet cell. The retentate was returned to the feed container. As the process was completed, the membrane unit was drained quickly and rinsed with RO water. The solution of Tergazyme enzyme‐containing detergent (Alconox, Inc., New York, NY) was prepared (2 g/L) and circulated through the system for 30 min. Once the system was fully drained, it was flushed with reverse osmosis (RO) water. The circulation of water was halted once the initial water volume was replenished; therefore, the membrane system was cleaned completely and the membrane was preserved in a solution containing 1% formaldehyde for future utilization (Tzeng, Diosady, and Rubin 1990).

### 2.1 Extraction of Proteins

The extractability of proteins from pinto bean flour was assessed across a pH range spanning from 2 to 12 according to the methods of Marnoch and Diosady (2006). For this purpose, 10 g portions of bean flour were added into pH‐adjusted aqueous NaOH solution at a liquid‐to‐solid ratio of 18 with mixing for 1 h at room temperature at each pre‐determined pH. While mixing, the pH level was continuously monitored and maintained at a constant value by gradually adding diluted acid or alkali as needed. Next, the obtained suspension was subjected to centrifugation at 9000 × *g*, 4°C for 30 min to separate the extract and solids. The supernatant was vacuum filtered through Whatman paper #541 (Prolabo, Bruchet Dano, Rennes, France). The residual solids were washed twice with water at a liquid‐to‐solid ratio of 6, and the resulting suspension was filtered each time through the filter paper and added to the extract solution. The amount of protein present in the supernatant was assessed using the AOCS standard method Ba 4d‐90vv (Lin, Krishnan, and Wang 2006), also known as the Kjeldahl nitrogen method. This method involved utilizing a Büchi model 425 digestion unit and a Büchi model K‐350 distillation unit. The protein's extractability was determined by calculating the weight ratio of the protein obtained from the initial feed solution per 10 g of the starting flour.

$$\text{Extractability}\;\left(\%\right)=\frac{\text{Grams of protein in the extract}\;}{\text{Amount of protein in}\;10\;g\;\text{of the initial flour}\;}\times 100$$

### 2.2 Precipitation of the Dissolved Proteins

In order to identify the ideal pH for protein precipitation and recovery, a protein feed solution weighing 400 g was prepared at a pH of 10.5, following the previously mentioned procedure. The pH of the solution was adjusted to set values in the pH range of 4–5.5 with 6 M H_3_PO_4_ and kept constant for 30 min. The suspension was centrifuged at 9000 × *g*, 4°C for 30 min to separate the precipitate. The precipitate was washed with water. The protein levels in both the washed precipitate and the filtrate were measured using the Kjeldahl nitrogen method, specifically, the AOCS method Ba 4d‐90 (Kumar et al. 2020).

The protein recovery was determined by calculating the ratio of the protein weight in the precipitate to the protein weight in the initial extract suspension.

### 2.3 Protein Recovery via Membrane Processing

**FIGURE 1:**
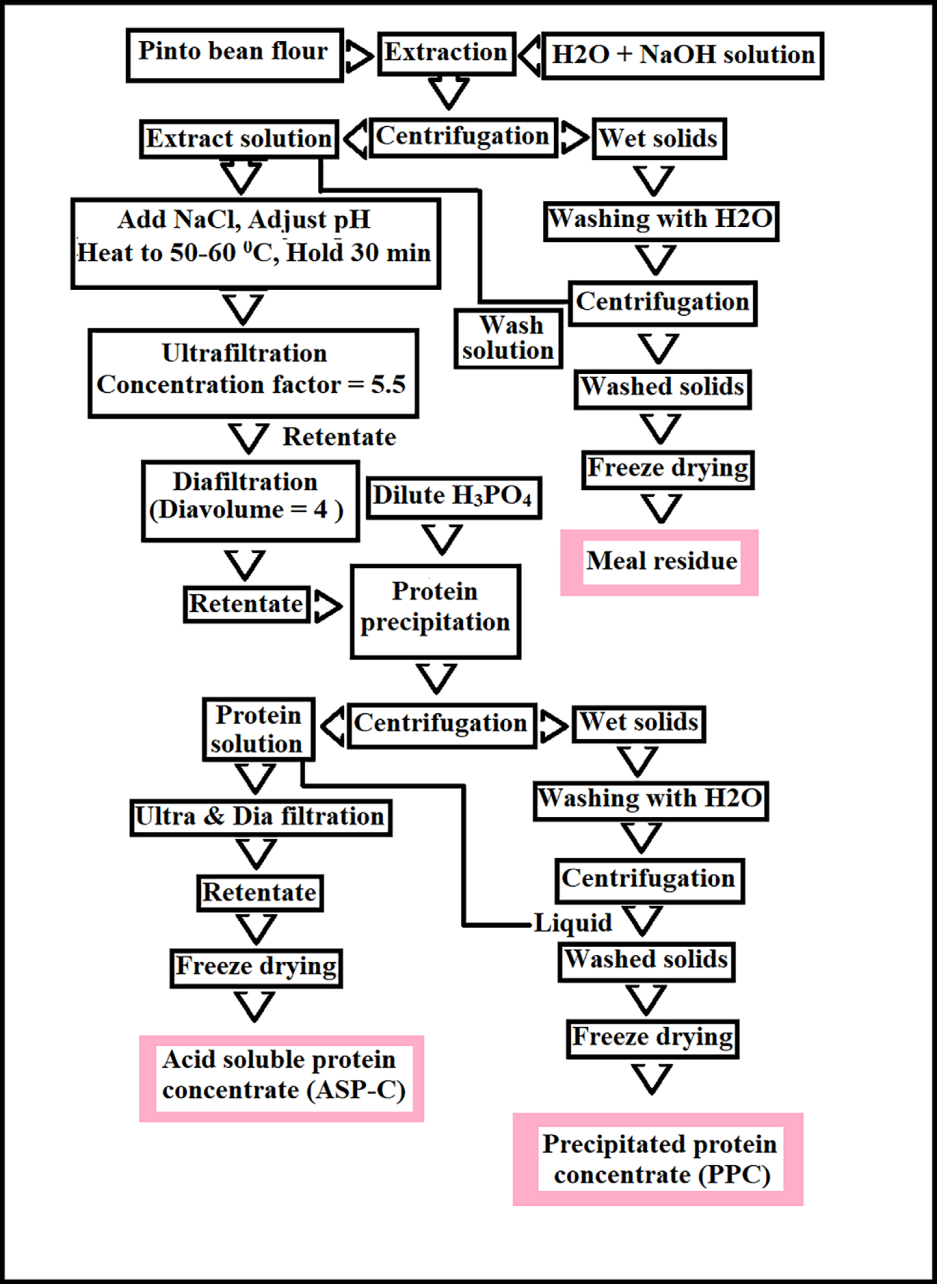
Process flow diagram for protein extraction from pinto bean flour.

A schematic diagram of the purification of pinto bean protein through membrane processing is presented in Figure 1. According to the methods of Kumar et al. (2020), pinto bean flour was mixed with water at a weight ratio of 18:1, and the pH was manually adjusted to 10.5 by adding 25% NaOH. The resulting suspension was stirred continuously at this pH for 1 h. Following the protein extraction process, any remaining solid particles were eliminated through centrifugation at 9000 × *g* and 4°C for a duration of 30 min. The solid materials were rinsed twice with water (weight ratio of 6) and then subjected to centrifugation in order to retrieve the extract. The solid residue was neutralized and subjected to freeze‐drying, while the wash solutions were consolidated with the initial extract. NaCl was added at 3 g/L and heated at 55°C for 30 min in order to dissociate phenolic compounds that are bounded to proteins through ionic bonding. The extract that underwent heat treatment was concentrated through ultrafiltration with a concentration factor of 5.5. It was subsequently purified through diafiltration using a polyethersulphone membrane with a molecular weight cut‐off of 10 kDa, with a dia‐volume of 4. The diafiltration process was performed by the addition of freshly prepared 0.05 M NaCl solution at pH 10.5 to the feed tank at the mass flow rate of permeate. The pH of the retentate was lowered to 4.5 by the addition of 6 M H_3_PO_4_. The isoelectric protein was precipitated and recovered by centrifugation. The moist precipitate was subjected to freeze‐drying in order to generate a concentrated protein product known as precipitated protein concentrate (PPC). The remaining transparent liquid portion, which contained proteins soluble in acid, underwent additional concentration and purification through ultrafiltration and diafiltration processes with a concentration factor (CF) and dia‐volume (DV) of 3. The resulting concentrated solution was freeze‐dried to produce acid‐soluble protein concentrate (ASP‐C). The process flow diagram is presented in Figure 1.

### 2.4 Chemical Analysis

The Kjeldahl method was utilized to determine the crude protein content, which was then calculated by multiplying the nitrogen content by a factor of 6.25, following the guidelines outlined in the AOCS Official Method Ba 4d‐90 (Thiex 2009). The total phenolics were evaluated using spectrophotometric analysis with Folin–Ciocalteu's reagent and were expressed as gallic acid equivalents (mg GAE/g) (Mehmood et al. 2018). Fat content was measured in triplicates by Soxhlet extraction with hexanes for a period of 24 h according to the AOAC method No. 963.15 (Thiex 2009). Moisture was evaluated gravimetrically and expressed as a percent of dry weight (Thiex 2009).

### 2.5 Functional Properties

#### 2.5.1 The Nitrogen Solubility Index

The nitrogen solubility index (NSI) was determined using the procedure described by John et al. (2021). The feed solution containing 0.5 g of sample in 20 mL of distilled water was mixed in a stirrer for 2 h. The resulting suspension was centrifuged at 9000 × *g*, 4°C for 30 min. The supernatant was separated, and its nitrogen content was analyzed using the Kjeldahl method. NSI was determined based on this formula:

$$\mathrm{NSI}\;\left(\%\right)=\frac{\%\text{Water soluble nitrogen}}{\%\text{Total nitrogen}}\times 100$$

#### 2.5.2 Water and Oil Holding Capacity

The water and oil holding capacity of the recovered proteins (PPC and ASP‐C) were determined based on the method of Ghodsvali, Khodaparast, Vosoughi, and Diosady (2005). For this purpose, 0.1 g of the sample was vortexed thoroughly (2 min) with 1.5 mL of distilled water or soybean oil in a weighed microcentrifuge tube. Then, the sample was kept at room temperature for half an hour, which was followed by centrifugation at 6000 × *g*, 4°C for 25 min. The resulting supernatant was removed, and the remaining solid was weighed. The water holding capacity (WHC) and oil holding capacity (OHC) were determined by quantifying the amount of water or oil in grams per gram of the sample.

#### 2.5.3 Emulsifying Activity and Stability

The EA was measured according to Dalev and Simeonova (1995) method with some adjustments. 3.5 g of samples was diluted in 50 mL water and homogenized at 10,000 rpm (Polytron homogenizer, Brinkmann, Westbury, NY). Emulsions were prepared with the same volume of canola oil through homogenization for 2 min. Then, the prepared emulsions were poured evenly into two 50 mL falcons and centrifuged at 2600 × *g*, 4°C for 5 min. The emulsifying activity was calculated using this formula:

$$\mathrm{EA}\;\left(\%\right)=\frac{\mathrm{EPV}}{\mathrm{WV}}\times 100$$

Here, EPV and WV represent the level of the emulsified layer (mL) and the initial level of emulsion before centrifugation (mL), respectively.

Emulsion stability (ES) was measured according to the method of Naczk, Diosady, and Rubin (1985) using the sample which was made to measure emulsifying activity. For this purpose, the sample was heated to 85°C for 15 min and after cooling was evenly split into two 50 mL falcon tubes. Next, they were centrifuged at 1100 × *g* for 15 min, and the emulsion stability was determined by calculating the percentage of remaining emulsifying activity.

#### 2.5.4 Whippability and Foam Stability

To measure whippability and foam stability, a 3% dispersion (50 mL) of samples was prepared in distilled water and then homogenized (Lin, Humbert, and Sosulski 1974). Homogenized samples were promptly transferred to a graduated cylinder with a volume of 250 mL, and the foam volume was recorded. The percentage of increased volume was considered as the whippability. Foam stability was calculated from the remaining foam volume after 0.5, 20, 40, 60, and 120 min with the base of foam volume of 200 mL of a 3% dispersion. The tests for functional properties were conducted under conditions of neutral pH.

#### 2.5.5 Statistical Analysis

The experiments were all repeated three times. The data were analyzed by SPSS 13.0 statistical software and expressed as mean ± SD (means ± standard deviation). One‐way ANOVA was completed by the post hoc Duncan's test at *p* < 0.05 to identify the significant differences between the means (*p* ≤ 0.05). All the figures were created using Origin Pro 2019.

## 3 Results and Discussion

### 3.1 Protein Extractability

**FIGURE 2:**
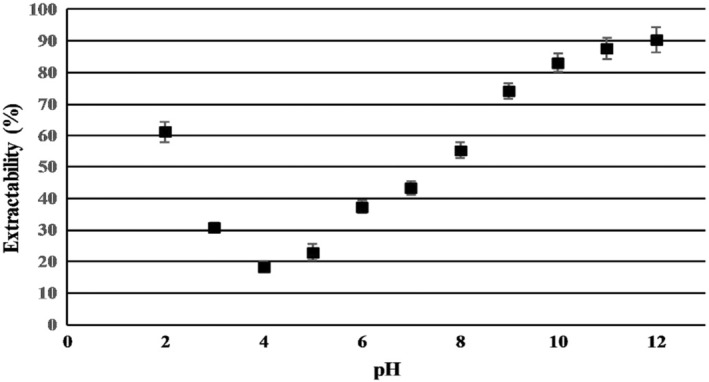
Effect of pH on protein extractability of pinto bean flour. Data represents the means of three replicates ± SD.

The pinto bean protein extractability in the pH range from 2 to 12 is presented in Figure 2. Studies on the extractability of protein from pinto beans demonstrated that the protein could be extracted effectively under both acidic and basic pH conditions but it was higher in the alkaline region than that was obtainable in the acidic region. The lowest protein extractability, 18.4%, was obtained near pH 4 (near to isoelectric point of protein [4.5]). There was a sharp rise in protein extractability when the pH was lowered from 3 to 2 as well as when it was increased from 5 to 9. Protein extractability increased slowly above pH 10. The highest extractability, 90.37%, was achieved at pH 12. The increased solubility of pinto bean protein at pH levels both above and below the isoelectric point (IEP) may be attributed to the predominant charge of the amino acids present in the proteins. The pH, which significantly influences peptide charge according to Cui et al. (2020), is a key factor in this phenomenon. At elevated pH levels, all the amines lose their protons, resulting in a net negative charge, whereas the opposite occurs at low pH levels. Protonation or deprotonation can improve the interaction between proteins and solvents. The electrostatic repulsion between proteins with similar charges keeps them separated, promoting their interaction with the solvent and enhancing protein solubility. As pH increases, the net negative charge increases thus desegregation (solubility) progressively increases. However, near the isoelectric point (pH 4.5), the predominant dipolar species of pinto bean protein leads to minimal repulsion and increased protein–protein interaction, resulting in the formation of insoluble aggregates and decreased protein solubility (Damodaran and Kinsella 1986). The extractability of pinto bean protein at different pH was in the same range as reported by Tan, Ying‐Yuan, and Gan (2014) for extraction of pinto bean protein using the alkaline solubilization‐acid precipitation method. A similar pattern of protein extractability from Chilean Granado Bean (*P. vulgaris* L.) and quinoa seeds has been reported by Kumar et al. (2020); (Liu et al. 2023) which subsequently increased the pH increased protein solubility progressively. While higher solubility was obtained at higher pH, extraction of protein above pH 11 was found to be impractical since the ultrafiltration membrane was not stable beyond pH 11. It has been proved that the use of a high alkaline pH during the extraction process can potentially disperse or dissolve non‐protein components like fiber and starch into the extraction suspensions, thereby lowering the overall purity of the protein (Kamani et al. 2023). Furthermore, it was demonstrated that protein undergoes a gradual breakdown and change into smaller subunits due to the presence of a high alkaline pH (pH > 11) and lysinoalanine formation at high pH values could render the isolate toxic (Jarpa‐Parra et al. 2014; Xu et al. 2023). As stated by Tan, Ying‐Yuan, and Gan (2014), the presence of numerous acidic amino acids, such as aspartic acid and glutamic acid, in pinto bean protein results in the generation of additional negative charges and strong repulsive forces between protein molecules that leads to the excellent solubility above pH 6. While a slight increase in protein extractability was observed at higher pH levels, pH of 10.5 with protein extractability of 86.05 ± 1.07 was chosen to avoid any potential damage to the protein caused by alkaline conditions.

### 3.2 Dissolved Protein Precipitation

**FIGURE 3:**
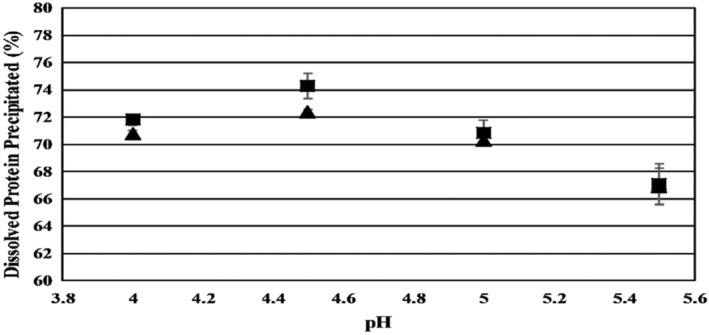
The amount of precipitated protein in the protein feed solution at varying pH. (▲) Measured from the protein content of precipitate; (■) Measured from the mass balance of protein in combined supernatant. Data represents the means of three replicates ± SD.

The isoelectric point of most legume proteins has been reported to be in the pH range of 4.0–5.3 (Contardo, Guzmán, and Enrione 2023; Fernández‐Quintela et al. 1997). Therefore, the pH range of 4–5.5 was selected to test the amount of precipitated protein. The amount of proteins that precipitated was determined by calculating the percentage of protein in the precipitate phase using the Kjeldahl method. As shown in Figure 3, the highest amount of precipitated protein (72.85%) was obtained at pH 4.5. Of the total crude protein content in the starting feed solution, 78%–85% was recovered as precipitated protein, which can be considered a promising yield. Kumar et al. (2020) reported the highest amount of precipitated protein of Chilean Granado bean (*P. vulgaris* L.) near pH 4 indicating a slightly lower isoelectric point. Additionally, in the research conducted by Chasquibol et al. (2022), the pH range of 4–5 was found to be the isoelectric point for red and gray beans.

### 3.3 Protein Products

The protein isolate can be defined as having at least 90% protein, while protein concentrates are defined as having < 75% protein (Hojilla‐Evangelista et al. 2014). In several studies, ultrafiltration (UF) has been recognized as a suitable method for efficiently recovering a significant amount of high‐quality plant‐based proteins (Dabestani, Arcot, and Chen 2017; Strætkvern and Schwarz 2012). Protein concentrates were obtained from batches of 80 g of ground pinto bean flour by following the procedure presented in Figure 1. The yield and protein content in the final products are presented in Table 1. About 17.3% of the bean flour was recovered as PPC, 3.9% as ASP‐C, and 54% as meal residue. Approximately 14.5% of the nitrogen was lost in the process. The un‐recovered protein consisted of non‐protein nitrogen, small peptides, and some transfer losses. These values are typical of losses in similar laboratory processes (Bhattarai, Al‐Ali, and Johnson 2022; Kumar et al. 2020). The mass recovery of the protein products (17.3 + 3.9) in this case was approximately in the same range of previous studies about NaOH‐extracted granado beans and CH_3_OH/NH_3_/H_2_O‐hexane‐extracted canola meal, which resulted in 22.5% and 29.6% mass recovery as protein products, respectively (Kumar et al. 2020; Xu et al. 2003). However, it was higher than the mass yield of green coffee protein concentrate (19.27 ± 0.30) prepared through alkaline extraction/ultrafiltration technique (Bhattarai, Al‐Ali, and Johnson 2022). This discovery emphasizes that the isoelectric precipitation process results in greater protein losses, as certain protein types (such as globulins) may not precipitate under acidic conditions. However, in the ultrafiltration process, all proteins should be retained in the retentate.

**TABLE 1:** Yield and protein content among products after membrane processing.^a^

Sample	Yield (%)	Protein content (%)
PBF	—	23.9 ± 0.11
PPC	17.3 ± 1.85	86.4 ± 0.09
ASP‐C	3.9 ± 0.21	56.3 ± 0.21
MR	54 ± 3.08	6.1 ± 0.10
Un‐recovered	24.8 ± 2.11	—

Abbreviations: ASP‐C, pinto bean acid soluble protein concentrate; MR, meal residue; PBF, pinto bean flour; PPC, pinto bean precipitated protein concentrate.

^a^ Data represents the means of three replicates ± SD.

The protein concentration of the samples was assessed using the Kjeldahl method and expressed as Nx6.25, which is a common technique in the food industry. Therefore, the starting protein value included non‐protein nitrogen compounds as protein. The yield of PPC was much higher than that of ASP‐C which was consistent with the results of previous studies (Diosady, Xu, and Chen 2007; Kumar et al. 2020). The relatively low protein content of ASP‐C in comparison with other plant proteins such as mustard and canola was extracted by a similar process and may be the consequence of leaching of some high‐molecular weight substrates such as starches into the extract solution (Kumar et al. 2020; Marnoch and Diosady 2006). In contrast to mustard, pinto beans have a notable amount of soluble starch with a high molecular weight. The membrane would capture and retain this starch during the process, allowing for its recovery in the retentate. As a result, the presence of this starch would dilute the fraction of acid‐soluble proteins (Kumar et al. 2020).

**FIGURE 4:**
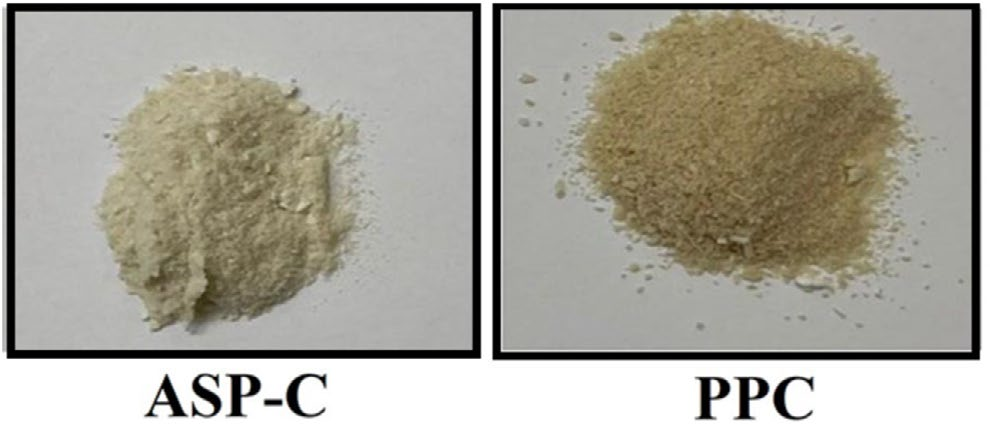
Acid soluble protein concentrate (ASP‐C) and precipitated protein concentrate (PPC) recovered from pinto bean glour.

The protein, fat, and polyphenol content of pinto bean flour and protein products are presented in Table 2. Both isolates were light in color but some differences in their appearance were observed (Figure 4). The PPC was sandy, while ASP‐C appeared fluffy. Based on the total phenolic values of the starting flour and recovered PPC protein in Table 2, the membrane process removed 74.26% of the phenolic compounds. This result was consistent with the result of Xu and Diosady (2002), which applied a membrane system for quinoa protein extraction and effectively recovered the phenolic compounds present in the starting flour. Furthermore, a study conducted by Hamzah and Leo (2023) demonstrated that ultrafiltration (UF) membranes are better suited for separating phenolic compounds from plants. Several studies have demonstrated the potential benefits of using ultrafiltration technology for protein extraction to enhance functional properties. Zhang et al. (2019) developed an effective method utilizing membrane technology to extract protein from the skim fraction, a byproduct of soybean oil extraction. The extracted protein exhibited an amino acid profile comparable to that of soy protein concentrate, with significantly lower amounts of trypsin inhibitors and phytate. This indicates that the extracted protein is of high quality. In conclusion, ultrafiltration (UF) can effectively extract high‐value protein from enzyme‐assisted extraction (EAE) of soybeans while removing undesirable components from the final protein product.

**TABLE 2:** Composition of pinto bean flour and protein products.^a^

Sample	Moisture %	Protein % (dry‐basis)	Fat %	Total phenol mg gallic acid/g sample
PBF	7.49 ± 0.28	23.9 ± 0.23	1.54 ± 0.13	16.05 ± 0.19
PPC	5.21 ± 0.31	86.4 ± 0.51	—	4.13 ± 0.04
ASP‐C	8.52 ± 0.17	56.3 ± 0.48	—	—

Abbreviations: ASP‐C, pinto bean acid soluble protein concentrate; PBF, pinto bean flour; PPC, pinto bean precipitated protein concentrate; SPI, commercial soybean protein isolate (Supro 500E).

^a^ Data represents the means of three replicates ± SD.

### 3.4 Functional Characteristics

Functional properties determine the usefulness of a protein in food products (Smith 2000). Protein solubility is considered as a key functional property (Kinsella 1979). The functionality experiments were measured in triplicate. For functionality tests, the sampleˈs suspension was adjusted to neutral pH (see Table 3).

**TABLE 3:** Functional characteristics of protein products from pinto beans and commercial soybean protein isolate.^1^

Sample	pH	NSI (%)	Water‐holding capacity (g/g)	Oil‐holding capacity (g/g)	Emulsifying activity (%)	Emulsion stability (%)
PPC	7.13 ± 0.02	52.64% ± 0.71^c^	1.96 ± 0.05^b^	1.51 ± 0.05^b^	98.6 ± 1.53^a^	93.17 ± 4.11^a^
ASP‐C	7.22 ± 0.01	79.4% ± 0.43^b^	—	2.44 ± 0.06^a^	12.46 ± 3.83^c^	94.1 ± 4.22^a^
SPI	7.1 ± 0.02	83.61% ± 0.5^a^	2.66 ± 0.11^a^	1.46 ± 0.06^b^	68.3 ± 3.26^b^	96.5 ± 3.59^a^

Abbreviations: ASP‐C, pinto bean acid soluble protein concentrate; NSI, nitrogen solubility index; PPC, pinto bean precipitated protein concentrate; SPI, commercial soybean protein isolate (Supro 500E).

^1^ Data represents the means of three replicates ± SD. Values in the same column followed by different superscripts are significantly different at *p* < 0.05.

The nitrogen solubility index (NSI) is a quick test that is used to measure protein solubility of a protein product. Both protein products showed a high NSI values but significantly lower than that of the soy protein isolate (*p* < 0.05). The relatively high NSI value of PPC at neutral pH is attributed to the high amounts of aspartic and glutamic acids in the structure of the pinto bean protein (free carboxyl groups) that are in their ionized form at neutral pH (Tan, Ying‐Yuan, and Gan 2014). Therefore, increased repulsive forces between protein molecules caused higher solubility at neutral pH. Interestingly, acid soluble protein concentrate (ASP‐C) showed a higher NSI value (79.4%) at pH 7.22 which results in its wide application in food industry. The ASP‐C protein showed high solubility across a wide pH range from acidic to neutral. This characteristic makes it well‐suited for inclusion in carbonated soft drinks or milk. Consequently, it enables the production of safe and nutritious beverages for regions that typically face protein shortages. The NSI value of ASP‐C was in agreement with the results of Xu and Diosady (1994), where rapeseed ASP‐C showed a high NSI value in a wide pH range. As reported by Pedroche et al. (2004), the composition of the protein can also have a significant impact on the NSI value. While protein is the primary constituent of the extracted protein isolate or concentrate, some of its mass consists of other components such as carbohydrates. These carbohydrates can interact with proteins, potentially altering the net charge and/or hydrophobicity of the proteins, thereby influencing protein solubility. In a study by Alonso‐Miravalles et al. (2019), the solubility of alkaline extracted‐acid precipitated lentile protein was found to be lower than that of UF‐membrane extracted across the entire pH range. This difference can be attributed to the elimination of soluble proteins during the extraction process, resulting in a higher proportion of insoluble protein fraction in isoelectric precipitated protein, while these soluble proteins are preserved during membrane extraction process and increase the protein solubility in a wide pH range. In our study, the selected functional properties of the pinto bean proteins were tested at neutral pH while in Table 4, these measurements were conducted at the protein's isoelectric point. As anticipated, the nitrogen solubility index (NSI) of the precipitated protein isolates at isoelectric pH for all the reported plant proteins was extremely low. A low nitrogen solubility index indicates that the protein isolate has limited solubility at the pH of the measurement, which can restrict its application in certain food products. Conversely, acid soluble protein isolates fraction showed the highest NSI value and even higher than commercial soybean isolate at their isoelectric point.

**TABLE 4:** The functional properties of pinto bean proteins in comparison with other protein isolates extracted through membrane processing.

Material	The optimized process condition	Protein content% (dry basis)	pH of functionality measurement	NSI (%)	OHC (%)	WHC (mL/g)	EA (%)	ES (%)	Reference
Pinto bean (PPC)	Extraction at pH 10.5	86.4	7.13	52.64	151	1.96	98.6	93.17	Current study
Pinto bean (ASP‐C)	Extraction at pH 10.5	56.3	7.22	79.4	244	Not detectable	12.46	94.1	Current study
Yellow mustard (PPI)	Extraction at pH 11	80–90		1.4	—	—	—	—	Tabtabaei et al. (2017)
Yellow mustard (SPI)	Extraction at pH 11	90–100	5.5	39.5	—	—	—	—	Tabtabaei et al. (2017)
Chinese rapeseed (PPI)	Extraction at pH 12	99.9	6.5	2.8	258	1.98	56.1	99.4	Xu and Diosady (1994)
Chinese rapeseed (SPI)	Extraction at pH 12	91.2	6.5	95.3	514	Not detectable	10.8	111.1	Xu and Diosady (1994)
Oriental mustard (PPI)	Extraction at pH 11	96.0	5	—	—	—	—	—	Marnoch and Diosady (2006)
Oriental mustard (SPI)	Extraction at pH 11	72.0	5		—	—	—	—	Marnoch and Diosady (2006)
Chilean granado bean (PPI)	Extraction at pH 10	84	4	1.7	—	—	—	—	Kumar et al. (2020)
Chilean granado bean (SPI)	Extraction at pH 10	53	4	100	—	—	—	—	Kumar et al. (2020)
Commercial soybean isolate1 (Supro 500E)	—	95	4.2	82.8	—	—	—	—	Kumar et al. (2020)
Commercial soybean isolate1 (Supro 500E)	—	95	6.91	82.8	147	4.99	72	98.6	Xu and Diosady (1994)
Lentil protein isolate	Extraction at pH 7.5	93.7	7.5	43	224	3.96	—	—	Alonso‐Miravalles et al. (2019)
Yellow pea protein isolates	Extraction at pH 7.5	97.76	7.5	48	—	—	—	—	Taherian et al. (2011)

Abbreviations: EA, emulsifying activity; ES, emulsifying stability; NSI, nitrogen solubility index; OHC, oil‐holding capacity; WHC, water hoding capacity.

The water‐holding capacity of protein indicates the ability of a protein to trap water in the protein matrix. This attribute is advantageous, as the ability to retain moisture plays a role in the texture and mouth‐feel of food products (Kinsella and Melachouris 1976), but excessive water absorption may not always be beneficial, as a material with extremely high water absorption can absorb an excessive amount of water, potentially leading to dehydration of other components within the system (Xu and Diosady 1994). The water‐holding capacity (WHC) of PPC was 1.35 times less than that of soybean protein isolate (SUPRO 500E) (Table 4) which was similar to the precipitated chinees rapeseed protein isolate (Xu and Diosady 1994). While, Granado bean precipitated protein isolate exhibited a high water absorption capacity, comparable to soybean isolates (Kumar et al. 2020). The reason for the low water holding capacity of PPC can be ascribed to the existence of oil and the restricted accessibility of sites that bind water, particularly polar amino acids, on the protein's side chain groups, as demonstrated by Tan, Ying‐Yuan, and Gan (2014). Sathe, Deshpande, and Salunkhe (1982) research has demonstrated that removing fat from protein concentrates can greatly enhance the WHC values by potentially enhancing the quantity of water‐binding sites on the protein molecules that were previously impeded in a lipid‐rich environment. The water holding capacity of ASP‐C due to its high solubility was not detectable—as the protein dissolved in the added water. Ma et al. (2022) indicated that all of the studied pulse proteins (pea, faba bean and lentil) exhibited significantly lower water‐holding capacity (WHC) compared to the soy proteins. The water‐holding capacity (WHC) is affected by the presence of charged and polar amino acids, as well as the spatial arrangement of the amino acids. When a significant portion of the charged and polar amino acids is not readily accessible for water molecules on the protein's surface, it will lead to a decrease in the water‐holding capacity (WHC). According to the result of Tan, Ying‐Yuan, and Gan (2014), the water‐holding capacity (WHC) of pinto bean protein isolate extracted using traditional alkalin solubilization‐acid recipitation method was found to be relatively low at 1.65 g/g. This can be attributed to the high surface hydrophobicity of the pinto bean proteins, which restricts the interactions between the proteins and water. The oil‐holding capacity (OHC) of the plant protein isolates was lower than the water‐holding capacity, This outcome is expected since the proteins used are mostly hydrophilic molecules that contain a significant proportion of charged and polar amino acid residues (Tan, Ying‐Yuan, and Gan 2014). The oil holding capacity of PPC (1.51 g/g) was comparable to soybean protein isolate (1.46 g/g) (*p* > 0.05), while ASP‐C showed the highest oil holding capacity (2.44 g/g). Based on the our findings both PPC and ASP‐C exhibited remarkable abilities to absorb oil which could be advantageous in oil‐rich food formulations. This can be attributed to the sample's capability to physically capture oil through capillary attraction within its overall volume (Kinsella and Melachouris 1976). Additionally, the presence of hydrophobic amino acid side chains on the protein molecules' surface serves as the main binding sites for the hydrocarbon chains of lipids (Xu and Diosady 1994).

The emulsifying activity of a protein is related to its ability to unfold and stabilize the oil–water interface (Euston, Hirst, and Hill 1997). On the other hand, emulsion stability is generally considered as the ability of droplets to remain suspended in the dispersion state (Baysan et al. 2019). Emulsifying activity and emulsion stability (ES) of pinto bean protein products was measured and compared with soy protein isolate (SUPRO 500E) to evaluate their applicability as emulsifiers in different food products. As presented in Table 3, PPC had superior emulsifying activity to soybean protein isolate (*p* < 0.05); thus, it can be employed to hinder alterations in the structure such as merging, separation, clumping, or settling and can increase texture and palatability of the food system (Martin et al. 2018). In contrast, ASP‐C possesses extremely low emulsifying activity. This may be attributed to the high solubility of ASP‐C (Xu and Diosady 1994). All emulsions were stable (emulsion stability > 90%) and the impact of heating on emulsified layers of samples was not significant. It has been reported that proteins with hydrophobic side groups on their surface are easily adsorbed on the oil–water interface (Hoffmann and Reger 2014). Therefore, the higher emulsifying activity for precipitated pinto bean protein (PPC) may be related to the globulin fractions (vicilin) of this protein which has higher surface hydrophobicity than albumins (Tan, Ying‐Yuan, and Gan 2014). Furthermore the greater flexibility and high interfacial activity of vicilin than other globulins resulting in higher emulsifying activity for preicipitated pinto bean protein (PPC) (Zhou et al. 2022). Generally, the result indicated that pinto bean protein products could be effective emulsifiers for different food applications.

The foam‐forming properties of proteins are associated with their ability to diffuse to the interface between air and water, adhere to the interface, and reorient or undergo conformational changes at the interface in order to reduce surface tension. The expansion of foam is commonly affected by the protein's flexibility and its speed of binding at the boundary between air and water (Kilara et al. 1996).

**FIGURE 5:**
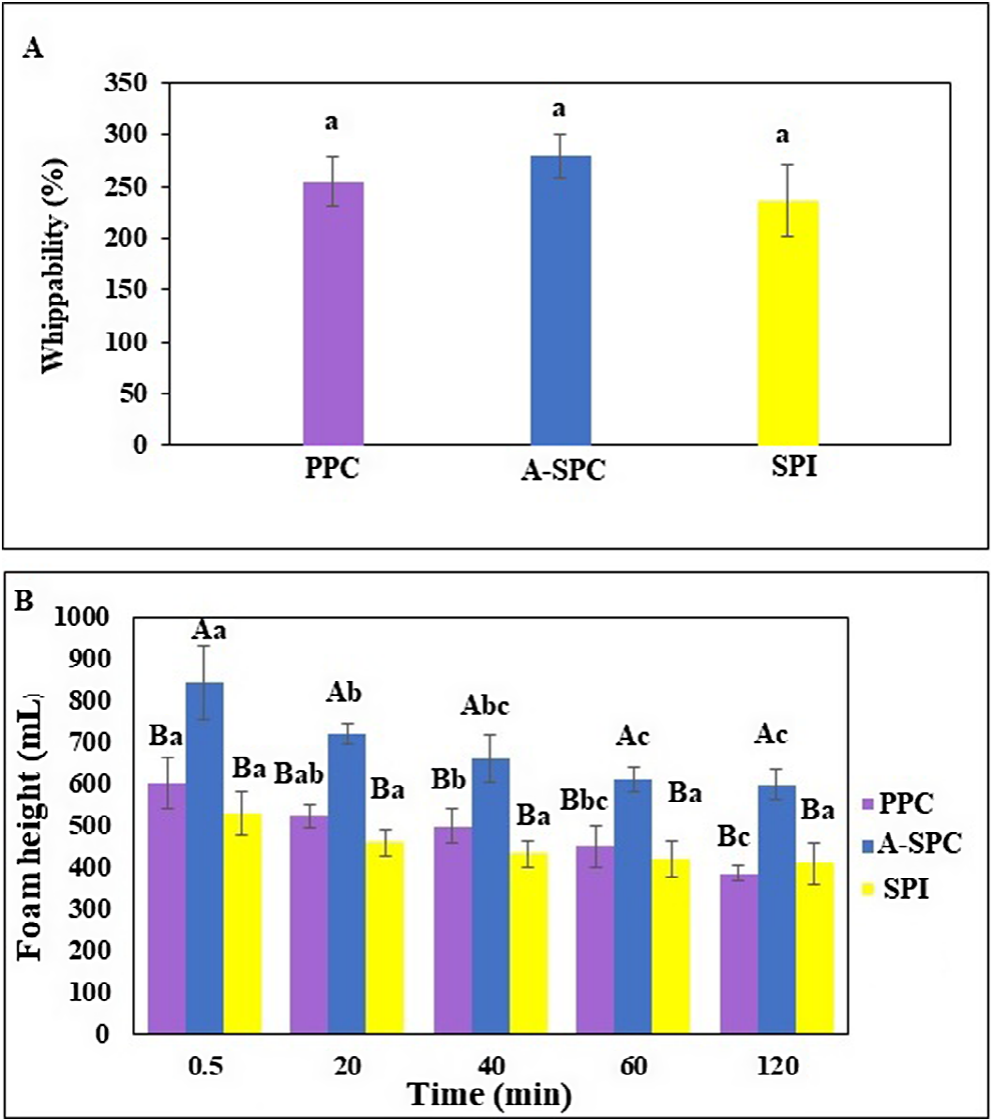
Foaming properties, whippability (A), and foam stability (B) of protein products from the pinto bean and soybean protein isolate. Different small letters indicate a significant difference at various time points for each sample (*p* < 0.05). Different capital letters demonstrate a significant difference among the various protein samples at a consistent time (*p* < 0.05). ASP‐C, pinto bean acid soluble protein concentrate; PPC, pinto bean precipitated protein concentrate; SPI, commercial soybean protein isolate (Supro 500E).

Data on the foaming properties of pinto bean protein products are reported in Figure 5.

Both pinto bean protein products had high whippability which was comparable with the soy protein isolate (Supro 500E) (*p* > 0.05). The stability of foams prepared from ASP‐C was significantly higher than PPC and SPI after all time intervals (*p* < 0.05). However, PPC and SPI did not show significant difference in foam stability in each time intervals (*p* > 0.05). The depletion of proteins with low molecular weight during the protein isolation process through membrane technology was believed to be the underlying factor responsible for the observed differences (Sosulski and McCurdy 1987). It has been proved that the foaming properties can be influenced by the isolation methods employed. Pea and horsebean proteins obtained through ultrafiltration of an alkaline extract exhibited higher foam capacity (FC) compared to commercial soy protein and skim milk powder (Vose 1980). On the other hand, proteins isolated through isoelectric precipitation had FC values lower than skim milk but higher than soy protein. During protein isolation procedures, the loss of albumins can have a negative impact on foaming properties. According to a study by Sathe and Salunkhe (1981), the albumins found in beans exhibited foam capacity (FC) values that were twice as high as the globulins, which had FC values similar to those of egg whites. Therefore, it can be anticipated that protein isolates containing albumins, obtained through ultrafiltration rather than isoelectric precipitation, would possess superior foaming properties. The foam stability of the ASP‐C and PPC foams decreased with passing time significantly (*p* < 0.05). This can be ascribed to the ability of the lipids present in this product to reduce foam formation (Yasumatsu et al. 1972). However, the stability of the foam prepared from soy protein isolate (Supro 500E) was constant in all the time intervals (*p* > 0.05). In a study conducted by Lee et al. (2007) using lentil proteins, it was reported that changes in protein conformation caused by increasing pH values during protein extraction had a significant impact on foaming properties. It was found that as the pH increased, the foam capacity (FC) decreased, indicating a decrease in the ability of the proteins to form stable foams. However, the foam stability (FS) increased, suggesting that the proteins had a greater ability to maintain their foam structure over time. This demonstrates that the conformational changes induced by pH variations can affect the foaming properties of lentil proteins.

## 4 Conclusions

The optimal conditions for recovering pinto bean protein via membrane processing were determined to be a pH of 10.5 for extraction, pH of 4.5 for precipitation, concentration factor of 5.5 for concentrating, and diavolume of 4 for purification. Under these conditions, two protein products PPC and ASP‐C with a protein content of 86.4% and 56.3% and the yield of 17.3% and 3.9% were successfully produced. Throughout this study, the remaining parameters, including the ratio of flour to solvent (1:18), extraction time (1 h), temperature (25°C), NaCl concentration (3 g/L), and heating time (55°C), were kept constant.

The functional properties of the products at neutral pH were comparable to commercial soy protein isolates. The precipitated pinto bean protein concentrate (PPC) demonstrated significantly superior emulsifying activity and foam stability to the soy protein isolate, making PPC a promising protein‐based emulsifier that can be used as a substitute of soy protein in various applications. Acid soluble protein concentrate (ASP‐C) demonstrated excellent nitrogen solubility index, making it suitable for specific applications in carbonated and acidic beverages. Based on this study, the utilization of a combination of alkaline extraction and membrane processing has the potential to disrupt the phenolic‐protein complexes, leading to the separation of the released phenolic compounds from the proteins. Therefore, while membrane processing may have a higher cost compared to the conventional acid precipitation method, it offers several environmental benefits and results in a protein product of superior quality with enhanced functional properties. In conclusion, by modifying the extraction and purification conditions, there is a possibility to broaden the range of functional properties exhibited by pinto bean protein isolate in different food systems, creating new possibilities and opportunities.

## Author Contributions

**Neda Aliabbasi:** writing – original draft (equal). **Levente L. Diosady:** conceptualization (equal), funding acquisition (lead), writing – review and editing (equal). **Zahra Emam‐Djomeh:** writing – review and editing (equal).

## Conflicts of Interest

The authors declare no conflicts of interest.

## Data Availability

All data underlying the results are available as part of the article, and no additional source data are required.
